# *Ninjin’yoeito* ameliorated PPE-induced pulmonary emphysema and anxiety/depressive-like behavior in aged C57BL/6J mice

**DOI:** 10.3389/fphar.2022.970697

**Published:** 2022-10-10

**Authors:** Taiki Shimoyama, Marisa Kaneda, Shota Yoshida, Seiwa Michihara, Nina Fujita, Li-kun Han, Ryuji Takahashi

**Affiliations:** Kampo Research Laboratories, Kracie Pharma, Ltd., 3-1 Kanebo-machi, Takaoka-City, Japan

**Keywords:** aged C57BL/6J, ninjin’yoeito, COPD, apoptosis, antioxidant, anxiety, depression

## Abstract

The prevalence of chronic obstructive pulmonary disease (COPD) is increasing in the elderly. COPD is a chronic respiratory disease characterized by airway remodeling and alveolar emphysema. COPD patients are also at high risk for mental illnesses such as depression and anxiety. *Ninjin’yoeito* (NYT) is prescribed to patients with conditions such as post-illness and postoperative weakness, fatigue, poor appetite, skin rash, cold hands and feet, and anemia. In addition to traditional uses, NYT is also prescribed as a therapeutic drug for poor functioning of the digestive organs, respiratory organs, and urinary organs. NYT is also known to have an antioxidant effect. The objective of this study was to investigate whether NYT could ameliorate COPD-induced lung injury and anxiety/depression in aged C57BL/6J mice exposed to porcine pancreatic elastase (PPE). While intratracheal administration of PPE induced emphysema in elderly mice, long-term administration of NYT suppressed the pathology. NYT was also found to suppress the apoptosis and oxidative stress caused by PPE. In addition, long-term administration of NYT was found to ameliorate PPE-induced depressive-like behavior in three different behavioral studies. These results suggest that NYT has a therapeutic effect on emphysema and the behavioral abnormalities caused by PPE.

## Graphical Abstract

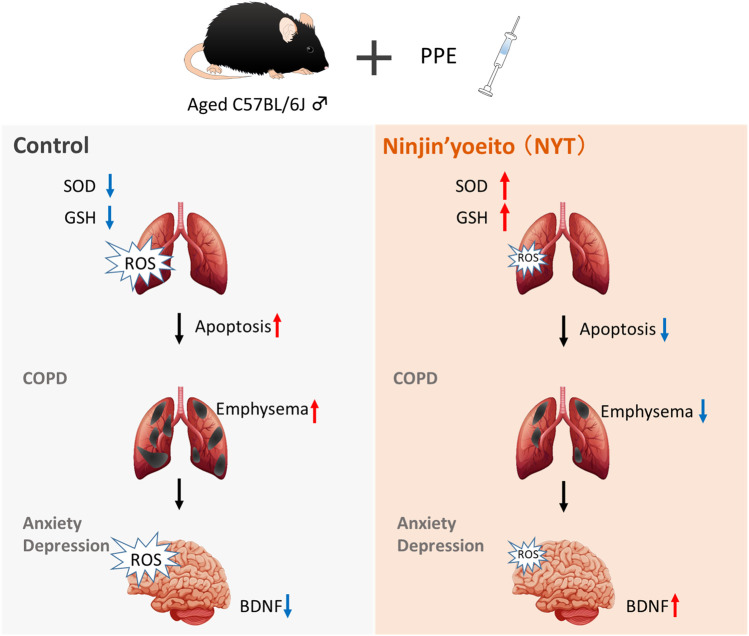

## Introduction

Chronic obstructive pulmonary disease (COPD), affecting approximately 12% of the worldwide population, is one of the main causes of death (Varmaghani et al., 2019). COPD is a lung disease caused by prolonged inhalation of noxious particles or gases such as air pollution or cigarette smoke. It is often accompanied by age-related comorbidities such as such as frailty, sarcopenia, and anxiety/depression (Barnes & Celli, 2009). COPD participants with frailty have higher mortality when compared with non-frail participants (Roberts et al., 2022). Anxiety and depression are well-recognized major comorbidities in COPD and reducing the negative effects of these comorbidities is important in the treatment of frail COPD (Cafarella et al., 2012; Wang et al., 2020). Worsening COPD pathophysiology is caused by a breakdown in the normal defense mechanisms of the body, and oxidative stress caused by cigarette smoke and air pollution is strongly implicated in this cause (Zinellu et al., 2016; Barnes, 2020). Oxidative stress occurs as a result of the increase in the reactive oxygen species (ROS) due to a decrease in endogenous antioxidants (Schieber & Chandel, 2014; Sies et al., 2017). This suggests that reducing oxidative stress by increasing the endogenous antioxidant activity may be an effective therapeutic approach to COPD pathophysiology. Multiple studies have described the therapeutic effect of antioxidants on respiratory diseases such as COPD (Biswas et al., 2013; Thomson, 2018).

*Ninjin’yoeito* (NYT) is a traditional Kampo (Japanese herbal medicine) listed as “Ho-chi-chü-fang” that consists of 12 types of herbs (Table 1). NYT is prescribed to patients with post-illness and postoperative weakness, fatigue, poor appetite, cold hands and feet, and anemia. In addition to these traditional uses, it has also been prescribed as a treatment for frailty of the gastrointestinal, respiratory, and urinary functions in recent years (Miyano et al., 2018). Recently, NYT treatment for elderly COPD patients has been reported to improve the reduction in the quality of life (QOL) as measured by the COPD assessment (Hirai et al., 2020). In addition, NYT therapy ameliorates the physical symptoms of COPD patients who are frail, such as weight loss and decreased muscle strength, and psychiatric symptoms such as depression and anxiety (Sakisaka et al., 2018; Hirai et al., 2020). Therefore, NYT is beneficial for both physical and psychiatric frailty symptoms. However, there are no reports confirming whether NYT improves COPD-related depression and anxiety in addition to emphysema *in vivo*. NYT has a dose-dependent effect of removing 1-Diphenyl-2-picrylhydrazyl radicals in rat blood plasma and a strong antioxidant effect (Egashira et al., 2003) that may be useful against COPD. Therefore, we hypothesized that, through the antioxidant action of NYT, it may ameliorate the pathophysiology of COPD as well as depression and anxiety. In the present study, we investigated whether NYT ameliorates lung injury and behavioral dysfunctions, such as depression and anxiety, in aged C57BL/6J mice with COPD.

**TABLE 1:** Medical herb composition/daily amount of *Ninjin’yoeito* (NYT).

Ingredients			Amount (g)
English Name	Latin Name	Original plant source and medicinal part	
Poria Sclerotium	*Poria*	The sclerotium of *Wolfiporia cocos* Ryvarden et Gilbertson (*Poria cocos* Wolf)	4
Japanese Angelica Root	*Angelicae Acutilobae Radix*	The root of *Angelica acutiloba* Kitagawa *or Angelica acutiloba* Kitagawa var. *sugiyamae* Hikino	4
Rehmannia Root	*Rehmanniae Radix*	The root of *Rehmannia glutinosa* Liboschitz var. *purpurea* Makino or *Rehmannia glutinosa* Liboschitz	4
Atractylodes Rhizome	*Atractylodis Rhizoma*	The rhizome of *Atractylodes japonica* Koidzumi ex Kitamura or *Atractylodes macrocephala* Koidzumi (*Atractylodes ovata* De Candolle)	4
Ginseng	*Ginseng radix*	The root of *Panax ginseng* C. A. Meyer (*Panax schinseng* Nees)	3
Cinnamon Bark	*Cinnamomi cortex*	The bark of the trunk of *Cinnamomum cassia* J. Presl	2.5
Citrus Unshiu Peel	*Citri Unshiu Pericarpium*	The pericarp of the ripe fruit of *Citrus unshiu* Marcowicz or *Citrus reticulata* Blanco	2
Polygala Root	*Polygalae Radix*	The root or root bark of *Polygala tenuifolia* Willdenow	2
Peony Root	*Paeoniae Radix*	The root of *Paeonia lactiflora* Pallas	2
Astragalus Root	*Astragali Radix*	The root of *Astragalus membranaceus* Bunge or *Astragalus mongholicus* Bunge	1.5
Schisandra Fruit	*Schisandrae Fructus*	The fruit of *Schisandra chinensis* Baillon	1
Glycyrrhiza	*Glycyrrhizae Radix*	The root and stolon of *Glycyrrhiza uralensis* Fischer or *Glycyrrhiza glabra* Linné	1

## Materials and methods

### Animal preparation and experimental protocol

Eighty-week-old male aged C57BL/6 mice (Charles River Laboratories, Yokohama, Japan) were used in these experiments. Animals were housed at 23 ± 2°C and 55 ± 10% under a 12-h light-dark cycle (lights on from 8:00 to 20:00) with *ad libitum* access to food and water. The behavioral experiments were performed between 12:00 and 18:00. All efforts were made to minimize the suffering and number of animals used. The experimental protocol was reviewed and approved by the Experimental Animal Care Committee of Kracie Pharma, Ltd. (Toyama, Japan).

### Plant materials

NYT extract powder (lot No. E1712111A0) was manufactured by the GMP Pharmaceutical Factory of Kracie Pharma, Ltd. (Qingdao, China). Each plant material quality was identified by external morphology and authenticated by marker compounds of the plant specimens (glycyrrhizic acid, paeoni florin, hesperidin, etc.) according to the method of the Japanese Pharmacopeia and the standards of Kracie Pharma, Ltd. NYT extract powder was mixed at 1% or 3% (w/w) with normal MF chow (Oriental Yeast Co., Ltd., Tokyo, Japan).

### High-performance liquid chromatography analysis of NYT

NYT extract (0.5 g) was mixed and shaken with 50% MeOH, and the supernatant was subjected to high-performance liquid chromatography (HPLC) analysis. The HPLC system (Nexera X3 system, Shimadzu Co., Kyoto, Japan) consisted of an SCL-40 system controller, LC-40B solvent delivery module, SIL-40C autosampler, CTO-40C column oven, SPD-M40 detector with a scanning range of 190–450 nm and a reversed-phase column (ACQUITY UPLC BEH C18 Column, 130 Å, 1.7 μm, 2.1 mm × 100 mm, Column temperature: 40°). The mobile phase consisted of 0.1% formic acid in water and 0.1% formic acid in MeOH. The flow rate was controlled by LC-40B at 0.3 ml/min.

### Drug treatment

Eighty-weeks-old male aged C57BL/6 mice were exposed to porcine pancreatic elastase (PPE; Sigma-Aldrich, St. Louis, MO, United States). The mice were divided into four groups: the control group, PPE-treated group, PPE + 1% NYT-chow group, or PPE + 3% NYT-chow group. PPE (4.0U/100 µl) or control (saline) was instilled into the tracheae using an intratracheal spray (21G song W103m, Natsume Seisakusho Co., Ltd., Japan. KN-34700-2), a mouse tracheal intubator (Muromachi Kikai Co., Ltd., Japan. MK-OS1), and an intubation stand. Subsequent to the PPE treatment, the mice were administered NYT chow consisted of 1% or 3% NYT powder mixed with MF chow for 20 weeks starting on the fourth-day post-injection. The control group received a regular diet of MF. Behavioral tests were started after 16 weeks of NYT treatment.

### Measurement of emphysema

The mouse lungs were fixed with 4% neutral buffered paraformaldehyde. The fixed lung was dehydrated, embedded in paraffin, sectioned, and stained with hematoxylin and eosin (H&E). Emphysema was quantified by measuring the mean linear intercept (MLI). Four randomly selected ×100 fields per specimen were photographed in a blinded manner. The MLI was obtained by dividing the length of a line drawn in the lung section by the total number of intercepts encountered in 20 lines per rat lung, as described previously (Thurlbeck, 1967).

### TUNEL assay

The level of apoptosis in the paraffin-embedded lung tissue from the mice was analyzed using an apoptosis *in situ* detection kit (Wako Pure Chemical Industries, Osaka, Japan) according to the manufacturer’s instructions, and the fluorescence-positive cells were photographed using an Axio Observer Inverted microscope (ZEISS). TUNEL-positive cells were counted in viable regions peripheral to areas in 500 cells within three randomly selected high power (×200) fields. The data were expressed as percentages.

### Oxidative stress-related enzymes

The right lung tissues were preserved in RNAlater^®^ (Thermo Fisher Scientific, Waltham, MA, United States) and homogenized with a Tissue Lyser LT (Qiagen, Hilden, Germany). The superoxide dismutase (SOD) activity was measured with a SOD Assay Kit-WST (Dojindo, Kumamoto, Japan), according to the manufacturer’s protocol. The total glutathione content in the lung homogenate was measured using the GSSG/GSH Quantification Kit (Dojindo, Kumamoto, Japan) according to the manufacturer’s instructions. The catalase (CAT) was measured using a CAT assay kit (Cayman Chemical Company, MI, United States). Each enzyme activity was analyzed using a microplate reader Synergy H1. All treatments were performed in triplicate.

### Open field test

The open field test was used to measure anxiety levels in mice. The procedure was carried out as described in the report by Seibenhener & Wooten (2015). Each mouse was placed at the periphery of the open field apparatus (width 30 cm × length 30 cm × height 30 cm). The total distance traveled in the arena or the number of crossing and the time spent the center zone (width 15 cm × length 15 cm) were recorded for 10 min using the video tracking system ANY-maze (Muromachi Kikai Co., Ltd., Japan).

### Tail suspension test (TST)

We performed the tail suspension test (TST) as described in a previous report (Can et al., 2012). Briefly, the tails of the mice were suspended with a piece of adhesive tape 50 cm above the floor with climbstoppers (clear plastic cylinder, 3 cm length, 1 cm outer diameter, 0.5 cm inner diameter), and the animal behavior was recorded for 6 min. As a test parameter, the total immobility time in the last 4 min was measured manually in a blinded manner. Small movements that were confined to the front legs without the involvement of the hind legs were counted as immobility. Additionally, oscillations and pendulum-like swings that were due to the momentum gained during the earlier mobility bouts were also counted as immobility.

### Forced swim test (FST)

The mice were placed in a glass cylinder (height, 30 cm; diameter, 15 cm) filled with water (26 ± 1°C) to a 15-cm depth for 6 min. The mice were judged to be immobile when they floated passively in the water, including only small movements to maintain their body balance or keep their heads above the water. As a test parameter, the mobility time during the last 4 min was measured manually in a blinded manner.

### Hippocampal BDNF

The left hippocampus tissues preserved in RNAlater^®^ were homogenized with a Tissue Lyser LT. The protein concentrations were determined using Pierce^®^ BCA Protein Assay Reagent (Thermo Lot. LI150452A) by mixing a 10-fold diluted protein extract with the assay solution containing bicinchoninic acid (BCA) and copper sulfate and reacting for 30 min at 37°C in a 96-well plate. Mature BDNF was measured using the Mature BDNF RapidTM ELISA kit (biosensis, BEK-2211) per the manufacturer’s instructions. Absorbance at 450 nm was measured on a microplate reader Synergy H1.

### Statistical analysis

All statistical analyses were performed using EZR (Saitama Medical Center, Jichi Medical University, Saitama, Japan), a graphical user interface for R-2.3-0 (The R Foundation for Statistical Computing, Vienna, Austria). The data were expressed as the mean ± standard error of means or median [25th percentile, 75th percentile]. Significant differences were assessed by a one-way analysis of variance followed by a Tukey test or Steel test for multiple comparisons. *p* < 0.05 was considered statistically significant.

## Results

### High-performance liquid chromatography analysis of NYT extract

**FIGURE 1:**
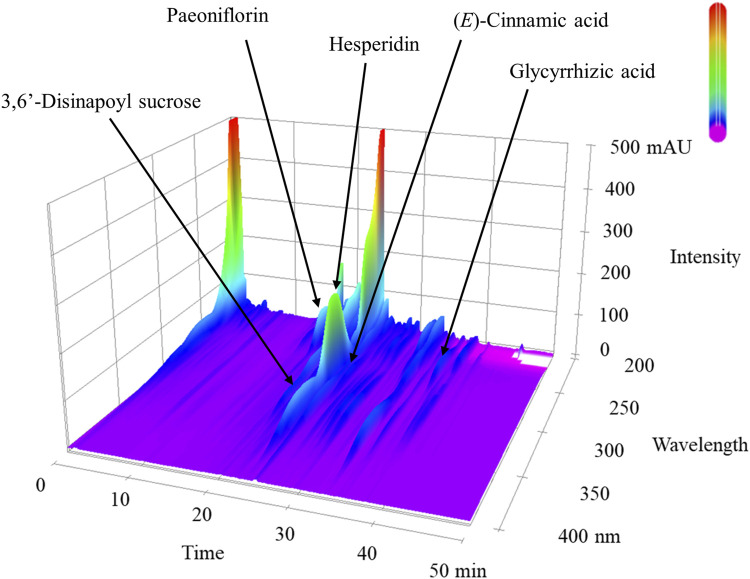
3D-HPLC profile of ninjin’yoeito (NYT). Each chemical marker (paeoniflorin, hesperidin, and glycyrrhizic acid, etc.) in the HPLC profile was identified by comparison with retention times and UV spectra of their reference standards.

The 3D-HPLC profile and chemical analysis of NYT are shown in Figure 1. The chemical manufacturers of paeoniflorin, hesperidin, glycyrrhizic acid, etc., were used as quality control.

### The efficacy of NYT treatment in PPE-induced emphysema

**FIGURE 2:**
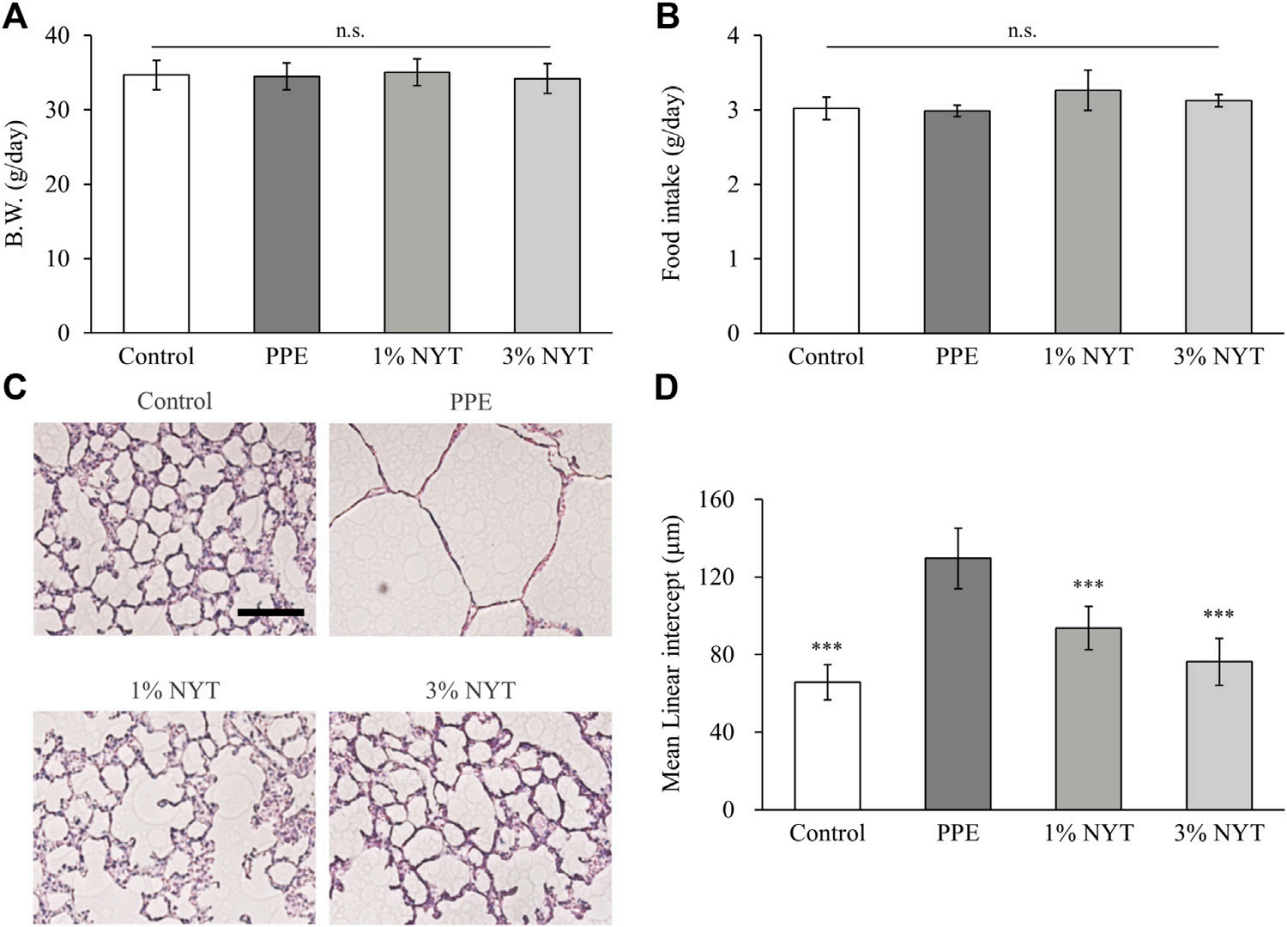
Effect of ninjin’yoeito (NYT) in lung tissue of PPE-induced COPD mice. **(A)** Average body weight and **(B)** food intake for NYT chow treatment term. **(C)** Lung histopathological analysis by hematoxylin and eosin stain. Scale bar = 100 µm. **(D)** Mean linear intercept of lung parenchyma. (Control *n* = 10, PPE *n* = 11, 1% NYT *n* = 8, 3% NYT *n* = 7). Values are expressed as means ± S.D. ∗∗∗*p* < 0.001 vs. PPE group by Tukey test.

**FIGURE 3:**
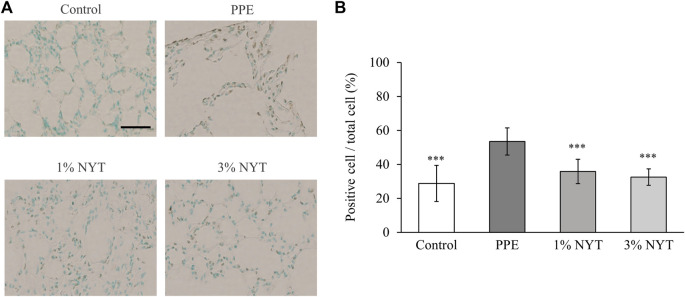
Effect of ninjin’yoeito (NYT) on lung tissue of PPE-induced apoptosis. **(A)** Lung sections stained by the TUNEL technique, counterstained with methyl green. Scale bar = 50 µm. **(B)** TUNEL-positive cell number expressed as percent of all nuclei counted. (Control *n* = 10, PPE *n* = 11, 1% NYT *n* = 8, 3% NYT *n* = 7). Values are expressed as means ± S.D. ∗∗∗*p* < 0.001 vs. PPE group by Tukey test.

No significant changes were observed in the mean body weight and amount of food intake between the groups during the feeding of NYT chow food (Figures 2A,B). We assessed the lungs of the aged C57BL/6J mice whose airways were exposed to PPE to investigate the protective effect of NYT on the lungs. The mean linear intercept (MLI: a measurement of alveolar/air space size) was utilized to show the alveolar phenotype associated with lung air space enlargement to assess the effect of PPE on alveolar morphology using HE staining. NYT was found to significantly suppress MLI enlargement caused by PPE (Figures 2C,D). TUNEL assay indicated apoptotic cells in pulmonary tissue, and the PPE-treated mice had a higher level of pulmonary apoptosis than the control mice. NYT was found to significantly reduce the PPE-induced lung apoptosis level (Figure 3). Thus, NYT is involved in the suppression of PPE-induced emphysema and lung apoptosis.

### The effect of NYT therapy on the antioxidant action in PPE-induced emphysema

**FIGURE 4:**
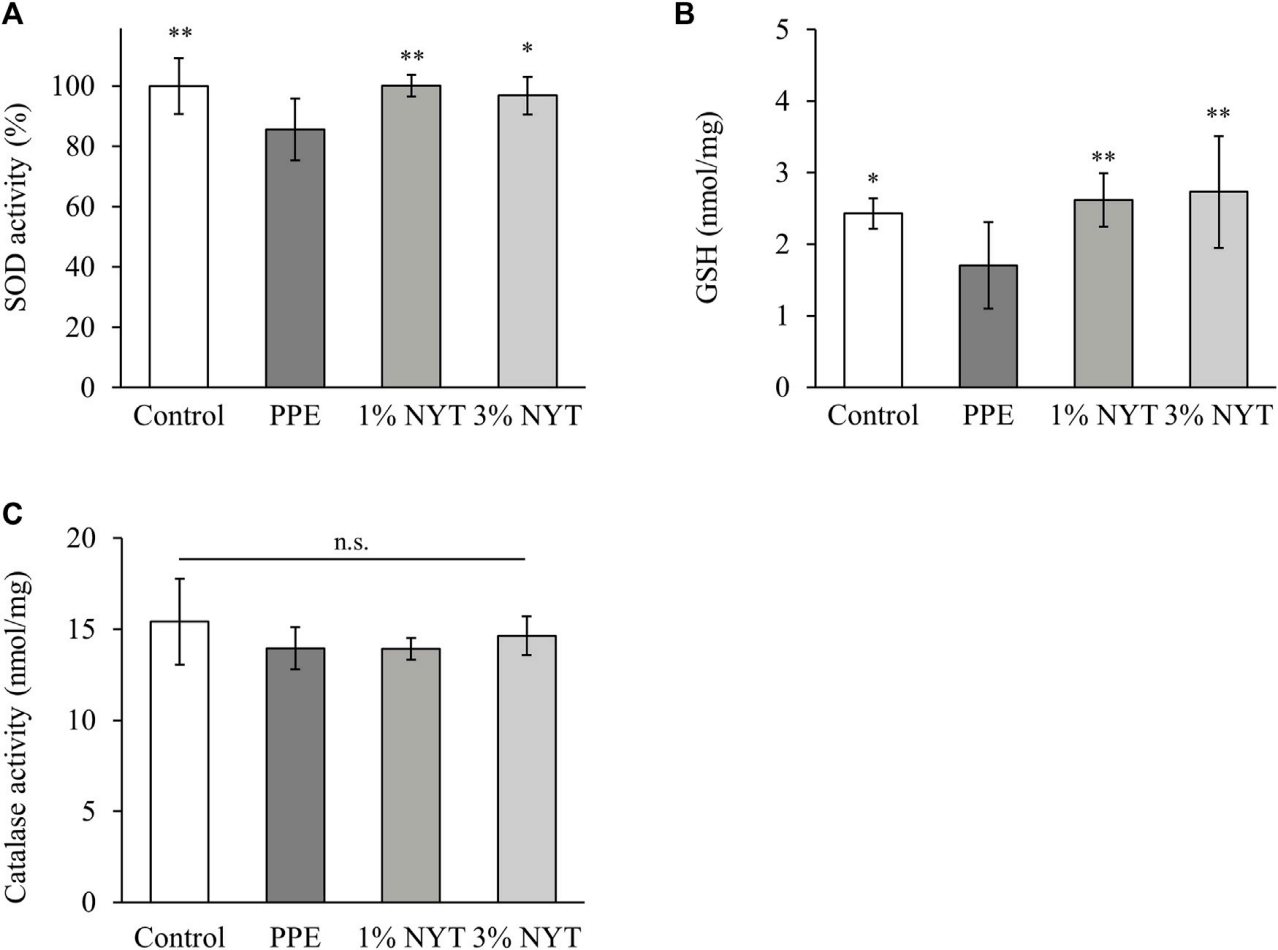
Effect of ninjin’yoeito (NYT) on lung tissue oxidative stress of PPE-induced COPD mice. **(A)** Total SOD activity, **(B)** level of GSH and **(C)** level of catalase in the lung homogenate. (Control *n* = 10, PPE *n* = 11, 1% NYT *n* = 8, 3% NYT *n* = 7). Values are expressed as means ± S.D. ∗*p* < 0.05, ∗∗*p* < 0.01 vs. PPE group by Tukey test.

The antioxidant action is an important factor in determining the efficacy of lung protection. We assessed the SOD activity as well as the GSH and catalase levels in a PPE emphysema model to investigate the antioxidant effect of NYT. PPE resulted in a decrease in the SOD activity and GSH level in the lung tissue. On the other hand, the SOD activity and GSH level significantly recovered as a result of NYT administration (Figures 4A,B). Catalase showed no significant inter-group changes (Figure 4C). These results suggest that NYT selectively promotes lung antioxidant factor activity.

### The effect of NYT therapy on PPE-induced behavioral abnormalities

**FIGURE 5:**
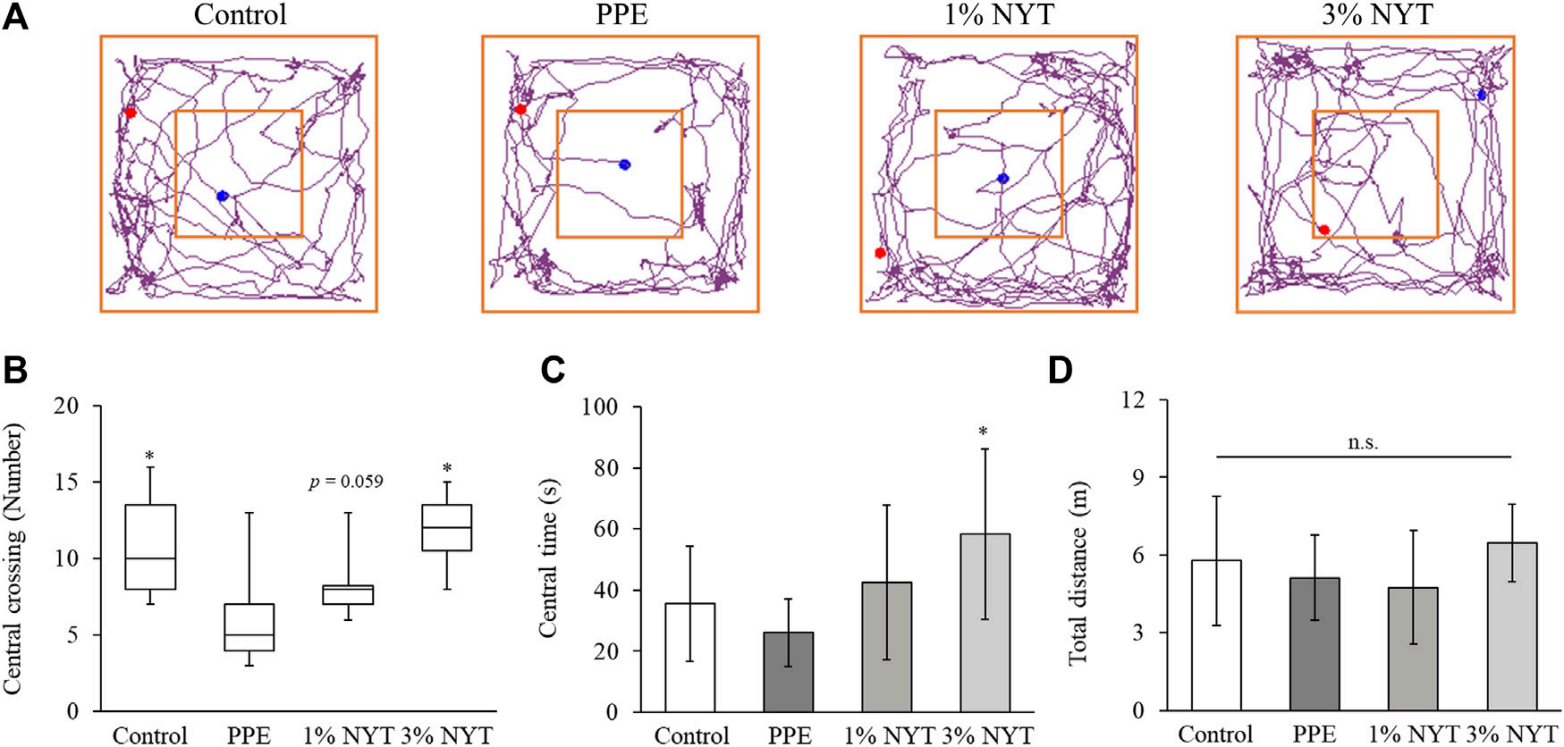
Effect of ninjin’yoeito on PPE-induced anxiety-like behaviors. **(A)** Tracing pathway, **(B)** Crossing number, **(C)** Time spent in the central zone and **(D)** Total distance in the open field. (Control *n* = 10, PPE *n* = 11, 1% NYT *n* = 8, 3% NYT *n* = 7). Values are expressed as median [25% tile, 75% tile] or means ± S.D. ∗*p* < 0.05 vs. PPE group by Steel or Tukey test.

We assessed the efficacy of NYT therapy on the anxious behavior in mice that were administered PPE. We utilized an open-field test for the assessment. While PPE administration reduced the number of times the mice entered the center zone, NYT administration resulted in a significant increase in the number of times the mice entered the center zone (Figures 5A,B). In addition, although PPE administration had no effect on the amount of time spent in the center zone, 3% NYT significantly extended the amount of time spent in the center zone (Figure 5C). No effect was found in either group on the total distance traveled (Figure 5D).

**FIGURE 6:**
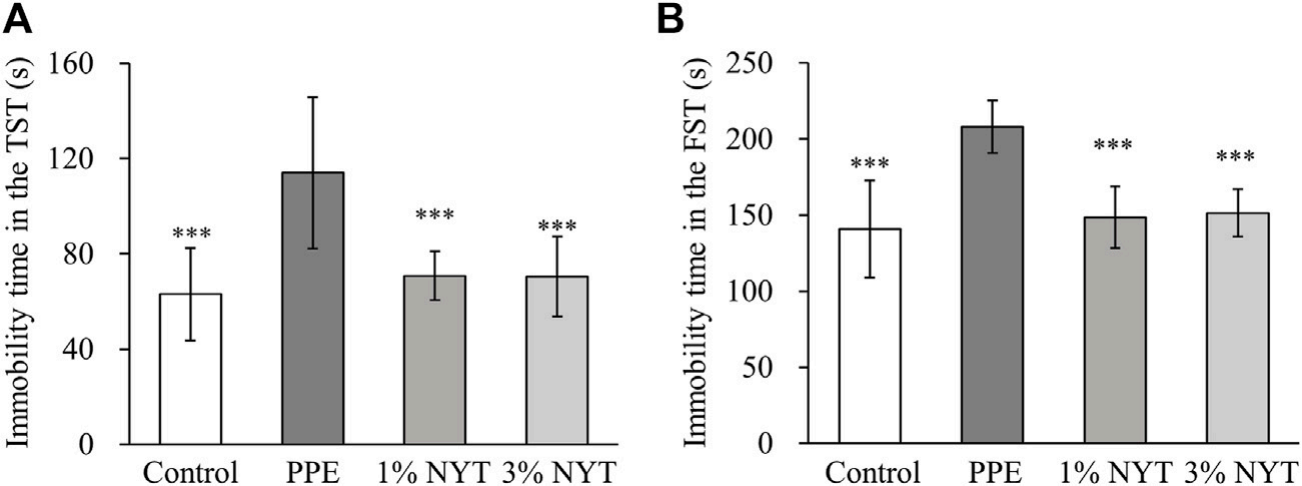
Effect of ninjin’yoeito (NYT) on PPE-induced depression-like behaviors. **(A)** Immobility time in the TST(s). **(B)** Immobility time in the FST(s). (Control *n* = 10, PPE *n* = 11, 1% NYT *n* = 8, 3% NYT *n* = 7). Values are expressed as means ± S.D. ∗∗∗*p* < 0.001 vs. PPE group by Tukey test.

**FIGURE 7:**
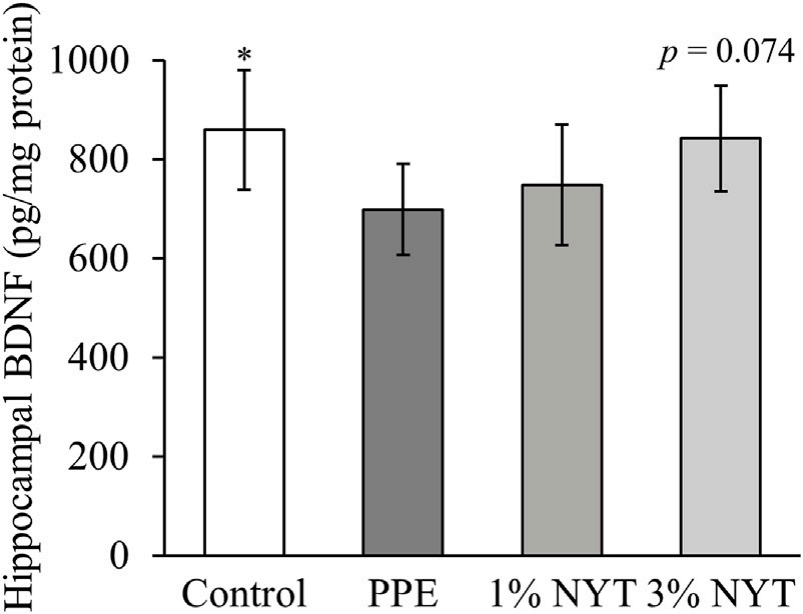
Effect of ninjin’yoeito (NYT) on hippocampal BDNF protein level in PPE-induced COPD mice. (Control n = 10, PPE *n* = 11, 1% NYT *n* = 8, 3% NYT *n* = 7). Values are expressed as means ± S.D. ∗*p* < 0.05 vs. PPE group by Tukey test.

Lastly, we assessed the effect of NYT therapy on the depressive-like behavior of mice that were administered PPE by performing the tail suspension test (TST) and the forced swim test (FST). In the TST, the PPE group showed a significantly increased amount of time spent immobile than that in the control group. In contrast, the NYT group showed a significant decrease in the amount of time spent immobile than that in the PPE group (Figure 6A). In the FST, NYT similarly showed a significant decrease in the amount of time spent immobile as a result of PPE (Figure 6B). We also measured the amount of BDNF, a factor related to depression, in the mice hippocampi. PPE significantly reduced the amount of BDNF, while NYT showed a trend of increasing the BDNF level (Figure 7). Therefore, we confirmed that NYT ameliorated PPE-induced anxiety and depressive-like behavior.

## Discussion

NYT treatment broadly ameliorated the PPE-induced emphysema in aged C57BL/6J mice that is associated with antioxidant action. In addition, NYT ameliorated the symptoms of anxiety and depression in aged C57BL/6J mice. To the best of our knowledge, this report is the first to investigate the long-term effect of NYT on aged C57BL/6J mice with COPD.

Assessment of alveolar injury caused by PPE in aged C57BL/6J mice showed that the MLI in mice that underwent long-term NYT treatment was suppressed to a significantly larger degree than that in the untreated mice (Figure 2). It has been reported in the past that short-term NYT treatment in young mice improved the destructive index resulting from exposure to cigarette smoke (Miyamoto et al., 2020). These results support the finding that NYT has a protective effect against alveolar injury in several COPD model mice. It is possible that the suppression of PPE-induced emphysema in mice treated with NYT may be due to the suppressive action on lung apoptosis (Figure 3). Apoptosis has been suggested as the mechanism for COPD emphysema formation. PPE-induced emphysema models are widely used in research as COPD model mice, and the pathogenesis mechanism is the promotion of apoptosis as a result of protease/anti-protease imbalance. Previous studies have reported that leukocyte elastase promotes apoptosis of the alveolar epithelial cells (Hou et al., 2013). The structural components of NYT have been shown to suppress apoptosis in several types of tissues, including that of the lungs (Zhang et al., 2008; Thenmozhi et al., 2017; Wen et al., 2018; Murata et al., 2022). An important piece of evidence that we found in our recent report was that NYT extract suppressed human pulmonary fibroblast apoptosis caused by cigarette smoke extract solution (Murata et al., 2022). NYT also has an antioxidant action on the mechanism that suppresses emphysema. It has been reported that the antioxidant activation factor in the bronchial lavage fluid of severe COPD patients decreases as the COPD pathophysiology becomes more severe (Drost et al., 2005). In the present study, the antioxidant activity in the lung tissue of PPE mice also decreased (Figure 4). Furthermore, similar to a previous report on the suppression of cigarette-induced emphysema by the promotion of antioxidant activity (Kubo et al., 2019), we found that the mice that were administered NYT showed increased antioxidant activity. Previously, it was frequently reported that NYT has a powerful antioxidant action (Egashira et al., 2003). In this mouse model, NYT improved the COPD pathophysiology via the suppression of apoptosis and promotion of the antioxidant effect.

Patients with COPD are highly likely to experience psychological symptoms such as depression and anxiety (Sievi et al., 2015). In a cigarette-exposure COPD model, hippocampus inflammation was found to manifest as COPD-related depressive-like behavior (Xie et al., 2019), and mental illnesses have been reported to occur clinically and *in vivo* in association with COPD. In the present study, long-term administration of NYT was observed to suppress the symptoms of PPE-induced anxiety and depression in COPD mice (Figures 5, 6). This result is consistent with the reports on NYT therapy ameliorating the anxiety and depression experienced by patients with COPD who are frail (Kuniaki et al., 2018; Hirai et al., 2020). In addition, skeletal muscle weights and spontaneous locomotor activity, such as that of the gastrocnemius and soleus muscles, did not change in this model (Supplementary Figures S1, S2). Therefore, we hypothesized that NYT acts on the psychiatric aspect and evaluated BDNF levels in the hippocampus. Patients with COPD with psychological symptoms such as anxiety and depression show decreased serum BDNF levels (Zhao & Dong, 2022). NYT mititgated the decrease in hippocampal BDNF in COPD mice, and this result is important to clarify the mechanism of improving anxiety and depression (Figure 7). BDNF, which plays an important role in maintaining brain functions, decreases as oxidative stress increases in cases of mental disorders. In addition to the increase in oxidative stress in the hippocampi of depression model mice, a decrease in the antioxidant activity and BDNF levels have also been reported (de Sousa et al., 2022), which suggests that there is a negative correlation between oxidative stress and BDNF levels. Although a detailed analysis of whether NYT controls oxidative stress in the brain remains necessary, antioxidant activity is thought to be one of the mechanisms by which NYT ameliorates anxiety and depression.

This study had several limitations. The first was the fact that it utilized aged C57BL/6J mice; thus, there is a need to conduct comparisons with young C57BL/6J mice. Mouse lung tissue has been reported to enlarge and become fragile with increasing age. In the present study, we did not identify whether NYT action has an effect only on more severe lung injuries. NYT is frequently used in clinical settings for frailty (Uto et al., 2018); thus, there is an expectation that it may have a systemic anti-aging effect. In the future, we plan to conduct an investigation that compares young and aged C57BL/6J mice. The second limitation was the fact that there is a need to conduct an assessment using brain tissue sections to analyze the mechanism by which NYT ameliorates mental disorders. We confirmed that NYT improves the decrease in Ki-67 and the number of DCX-positive cells in the hippocampi of corticosterone-induced depression model mice. However, in the present study, we were unable to confirm whether the maintenance of BDNF levels due to NYT administration had an effect on hippocampal neurogenesis. The dosage of NYT has been adjusted from a human clinical dose. The therapeutic dosage of NYT for human prescription authorized by the Ministry of Health, Labor and Welfare of Japan is 6.7 g/day. The adequate amount of NYT intake for aged mice was determined to be 1% NYT in this study; however, NYT, a traditional Kampo medicine, contains a variety of components, as shown by the HPLC results. Therefore, effective doses of NYT vary in different pathologies condition. Thus, further studies are necessary to identify the optimum dose-dependent mechanism in different pathologies by which NYT ameliorates lung injury and behavioral abnormalities.

In conclusion, the present study showed that NYT ameliorates PPE-induced lung injury via antioxidant activity. In addition, NYT was found to improve hippocampal BDNF levels and ameliorate anxiety and depressive-like behavior. Our findings suggest that the multifaceted effect of NYT is a novel prophylactic and therapeutic strategy for COPD or frail COPD.

## Data Availability

The original contributions presented in the study are included in the article/supplementary material, further inquiries can be directed to the corresponding author.

## Supplementary material

SUPPLEMENTARY FIGURE S1 Effect of NYT on muscle weight in PPE-induced mice. (A) Gastrocnemius muscle. (B) Soleus muscle (Control n = 10, PPE n = 11, 1% NYT n = 8, 3% NYT n = 7). Values are expressed as means ± S.D. ∗*p* < 0.05 vs. PPE group by Tukey test.

SUPPLEMENTARY FIGURE S2 Locomotor activities under light-dark cycle in PPE-induced mice. Mice were housed individually in transparent cages, kept on a 12-h artificial light/12-h dark cycle (dark on 20:00-08:00), and given food and water *ad libitum*. (A) Each value represents accumulative scores for 30 min (B–D Total locomotor activity for 24 h or 12 h (light or dark phase). Values are expressed as means or median [25th percentile, 75th percentile].

## References

[B1] Barnes P. J. Celi B. R. (2009). Systemic manifestations and comorbidities of COPD. Eur. Repir J. 33 (5), 1165–1185. 10.1183/09031936.00128008 19407051

[B2] Barnes P. J. (2020). Oxidative stress-based therapeutics in COPD. Redox Biol. 33, 101544. 10.1016/j.redox.2020.101544 32336666 PMC7251237

[B3] Biswas S. Hwang J. W. Kirkham P. A. Rahman I. (2013). Pharmacological and dietary antioxidant therapies for chronic obstructive pulmonary disease. Curr. Med. Chem. 20, 1496–1530. 10.2174/0929867311320120004 22963552

[B4] Cafarella P. A. Effing T. W. Usmani Z. A. Frith P. A. (2012). Treatments for anxiety and depression in patients with chronic obstructive pulmonary disease: A literature review. Respirology 17, 627–638. 10.1111/j.1440-1843.2012.02148.x 22309179

[B5] de Sousa C. Medeiros I. Vasconcelos G. S. de Aquino G. A. Cysne Filho F. de Almeida Cysne J. C. (2022). Involvement of oxidative pathways and BDNF in the antidepressant effect of carvedilol in a depression model induced by chronic unpredictable stress. Psychopharmacol. Berl. 239, 297–311. 10.1007/s00213-021-05994-6 35022822

[B6] Drost E. M. Skwarski K. M. Sauleda J. Soler N. Roca J. Agusti A. (2005). Oxidative stress and airway inflammation in severe exacerbations of COPD. Thorax 60, 293–300. 10.1136/thx.2004.027946 15790984 PMC1747355

[B7] Egashira T. Takayama F. Komatsu Y. (2003). Changes of materials that scavenge 1,1-diphenyl-2-picrylhydrazyl radicals in plasma by per-oral administration of Kampo medicine, Ninjin-yoei-to in rats. Pharm. Pharmacol. 55, 367–371. 10.1211/002235702711 12724043

[B8] Hirai K. Homma T. Matsunaga T. Akimoto K. Yamamoto S. Suganuma H. (2020). Usefulness of ninjin'yoeito for chronic obstructive pulmonary disease patients with frailty. J. Altern. Complement. Med. 26, 750–757. 10.1089/acm.2020.0083 32551796

[B9] Hou H. H. Cheng S. L. Liu H. T. Yang F. Z. Wang H. C. Yu C. J. (2013). Elastase induced lung epithelial cell apoptosis and emphysema through placenta growth factor. Cell Death Dis. 4, e793. 10.1038/cddis.2013.329 24008737 PMC3789187

[B10] Kubo H. Asai K. Kojima K. Sugitani A. Kyomoto Y. Okamoto A. (2019). Astaxanthin suppresses cigarette smoke-induced emphysema through Nrf2 activation in mice. Mar. Drugs 17, 673. 10.3390/md17120673 PMC6950584 31795292

[B11] Kuniaki H. Akihiko T. Tetsuya H. Hatsuko M. Tomoko K. Shin O. (2018). Improvement in frailty in a patient with severe chronic obstructive pulmonary disease after ninjin'yoeito therapy: A case report. Front. Nutr. 5, 71. 10.3389/fnut.2018.00071 30234120 PMC6131554

[B13] Miyamoto A. Asai K. Kadotani H. Maruyama N. Kubo H. Okamoto A. (2020). Ninjin’yoeito ameliorates skeletal muscle complications in COPD model mice by upregulating peroxisome proliferator-activated receptor γ coactivator-1α expression. Int. J. Chron. Obstruct Pulmon Dis. 15, 3063–3077. 10.2147/COPD.S280401 33273811 PMC7708308

[B14] Miyano K. Nonaka M. Uzu M. Ohshima K. Uezono Y. (2018). Multifunctional actions of ninjinyoeito, a Japanese Kampo medicine: Accumulated scientific evidence based on experiments with cells and animal models, and clinical studies. Front. Nutr. 5, 93. 10.3389/fnut.2018.00093 30349821 PMC6186795

[B15] Murata K. Fujita N. Takahashi R. (2022). Ninjinyoeito ameliorated cigarette smoke extract-induced apoptosis and inflammation through JNK signaling inhibition in human lung fibroblasts. BMC Complementary Med. Ther. 22, 96. 10.1186/s12906-022-03574-5 PMC8973640 35361188

[B16] Roberts M. H. Mapel D. W. Ganvir N. Dodd M. A. (2022). Frailty among older individuals with and without COPD: A cohort study of prevalence and association with adverse outcomes. Int. J. Chron. Obstruct Pulmon Dis. 17, 701–717. 10.2147/COPD.S348714 35411140 PMC8994612

[B17] Sakisaka N. Mitani K. Sempuku S. Imai T. Takemoto Y. Shimomura H. (2018). A clinical study of ninjin'yoeito with regard to frailty. Front. Nutr. 5, 73. 10.3389/fnut.2018.00073 30320119 PMC6165905

[B18] Schieber M. Chandel N. S. (2014). ROS function in redox signaling and oxidative stress. Curr. Biol. 24, R453–R462. 10.1016/j.cub.2014.03.034 24845678 PMC4055301

[B19] Seibenhener M. L. Wooten M. C. (2015). Use of the open field maze to measure locomotor and anxiety-like behavior in mice. J. Vis. Exp. (96), 52434. 10.3791/52434 PMC4354627 25742564

[B20] Sies H. Berndt C. Jones D. P. (2017). Oxidative stress. Annu. Rev. Biochem. 86, 715–748. 10.1146/annurev-biochem-061516-045037 28441057

[B21] Sievi N. A. Senn O. Brack T. Brutsche M. H. Frey M. Irani S. (2015). Impact of comorbidities on physical activity in COPD. Respirology 20, 413–418. 10.1111/resp.12456 25565363

[B22] Thenmozhi A. J. Raja T. R. W. Manivasagam T. Janakiraman U. Essa M. M. (2017). Hesperidin ameliorates cognitive dysfunction, oxidative stress and apoptosis against aluminium chloride induced rat model of Alzheimer's disease. Nutr. Neurosci. 20, 360–368. 10.1080/1028415x.2016.1144846 26878879

[B23] Thomson N. C. (2018). Targeting oxidant-dependent mechanisms for the treatment of respiratory diseases and their comorbidities. Curr. Opin. Pharmacol. 40, 1–8. 10.1016/j.coph.2017.11.013 29223018

[B24] Thurlbeck W. M. (1967). The internal surface area of nonemphysematous lungs. Am. Rev. Respir. Dis. 95, 765–773. https://www.atsjournals.org/doi/pdf/10.1164/arrd.1967.95.5.765 6023510

[B25] Uto N. S. Amitani H. Atobe Y. Sameshima Y. Sakaki M. Rokot N. (2018). Herbal medicine ninjin'yoeito in the treatment of sarcopenia and frailty. Front. Nutr. 5, 126. 10.3389/fnut.2018.00126 30619872 PMC6299011

[B26] Varmaghani M. Dehghani M. Heidari E. Sharifi F. Moghaddam S. S. Farzadfar F. (2019). Global prevalence of chronic obstructive pulmonary disease: Systematic review and meta-analysis. East Mediterr. Health J. 25, 47–57. 10.26719/emhj.18.014 30919925

[B27] Wang Z. Hu X. Dai Q. (2020). Is it possible to reverse frailty in patients with chronic obstructive pulmonary disease? Clinics 75, e1778. 10.6061/clinics/2020/e1778 33146351 PMC7561069

[B28] Wen H. Wu Z. Hu H. Wu Y. Yang G. Lu J. (2018). The anti-tumor effect of pachymic acid on osteosarcoma cells by inducing PTEN and Caspase 3/7-dependent apoptosis. J. Nat. Med. 72, 57–63. 10.1007/s11418-017-1117-2 28856634

[B29] Xie Y. He Q. Chen H. Lin Z. Xu Y. Yang C. (2019). Crocin ameliorates chronic obstructive pulmonary disease-induced depression via PI3K/Akt mediated suppression of inflammation. Eur. J. Pharmacol. 862, 172640. 10.1016/j.ejphar.2019.172640 31491407

[B30] Zhang G. Liu A. Zhou Y. San X. Jin T. Jin Y. (2008). Panax ginseng ginsenoside-Rg2 protects memory impairment via anti-apoptosis in a rat model with vascular dementia. J. Ethnopharmacol. 115, 441–448. 10.1016/j.jep.2007.10.026 18083315

[B31] Zhao N. Dong C. (2022). Correlation of serum IL-18, BDNF, and IL-1β with depression and prognosis after acute exacerbation of chronic obstructive pulmonary disease. Comput. Math. Methods Med. 2022, 3555982. 10.1155/2022/3555982 PMC9078809 35535228

[B32] Zinellu E. Zinellu A. Fois A. G. Carru C. Pirina P. (2016). Circulating biomarkers of oxidative stress in chronic obstructive pulmonary disease: A systematic review. Resp. Res. 17 (1), 150. 10.1186/s12931-016-0471-z PMC5109807 27842552

