# Supplementary figures and images for: Ninjin’yoeito ameliorated PPE-induced pulmonary emphysema and anxiety/depressive-like behavior in aged C57BL/6J mice
Source: Front Pharmacol. 2022 Oct 10;13:970697. doi: 10.3389/fphar.2022.970697 (PMC9589273; doi:10.3389/fphar.2022.970697)

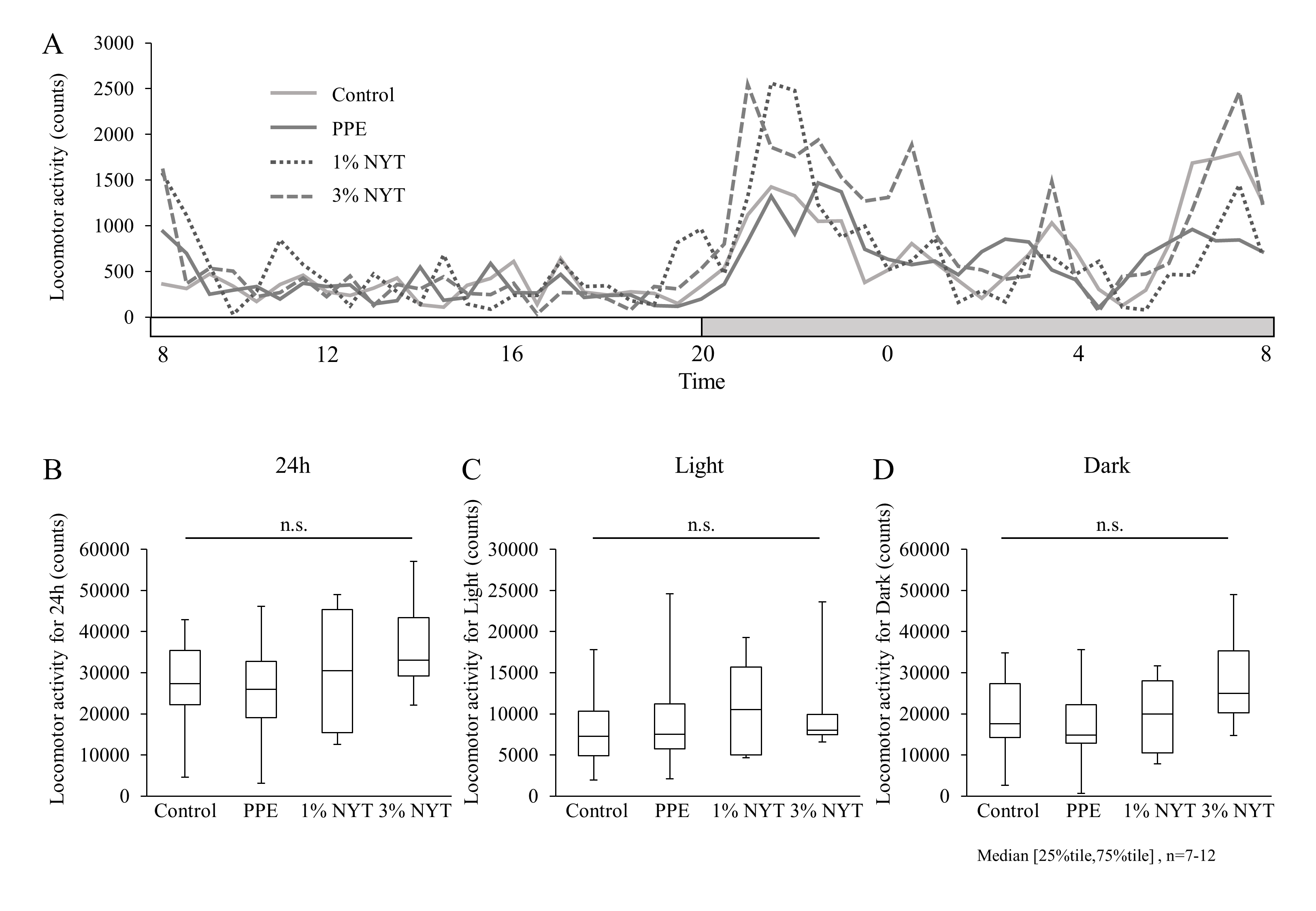

Supplement: Supplementary file 1 [file Image2.tif]

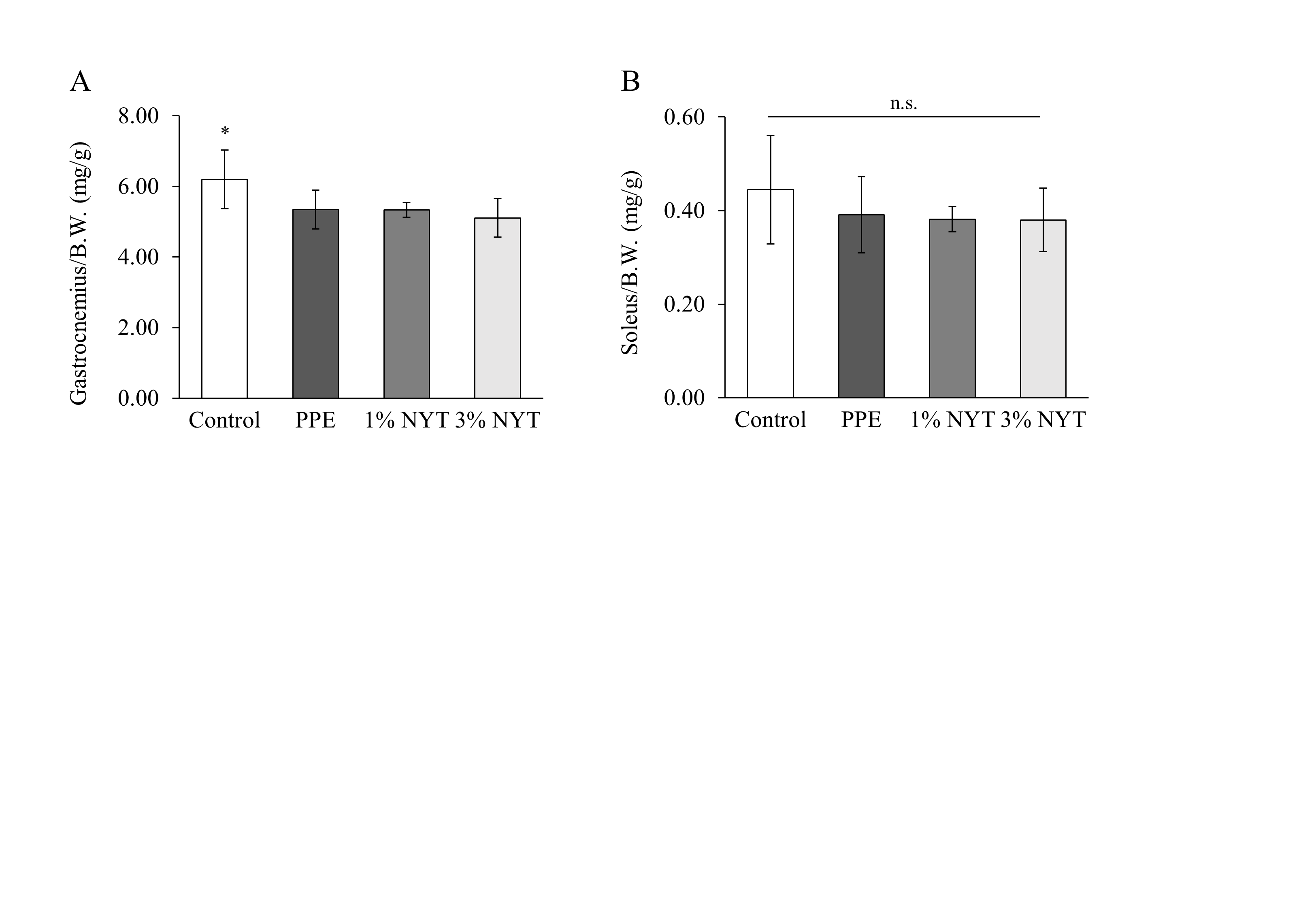

Supplement: Supplementary file 2 [file Image1.tif]
