# Supplementary material for: Ninjin’yoeito ameliorated PPE-induced pulmonary emphysema and anxiety/depressive-like behavior in aged C57BL/6J mice
Source: Front Pharmacol. 2022 Oct 10;13:970697. doi: 10.3389/fphar.2022.970697 (PMC9589273; doi:10.3389/fphar.2022.970697)
Supplement: Supplementary file 3 [file DataSheet1.PDF]

Supplemental Methods

1.1 Muscle weight

On the final day of the experiment, the mice underwent necropsy under anesthetic Isoflurane inhalation. Two types of muscles were excised, namely the gastrocnemius muscle and the soleus muscle, and their wet weights were measured.

1.2 Locomotor activity

The locomotor activity test was performed using a modification to procedures described previously (Yamada et al., 2019). Locomotor activity was monitored using an activity sensor unit for mice (Supermex PAT. P, Muromachi Kikai Co., Ltd., Tokyo, Japan). Each mouse was placed into an apparatus (30 × 20 × 13 cm), kept on a 12-hour artificial light/12-hour dark cycle (dark on 20:00-08:00), and given food and water ad libitum. Locomotor activity was measured for 24h and data were analyzed to determine the activity counts by Compact ASM3 software (Muromachi Kikai Co., Ltd.). Mice were acclimated to monitoring for 1 day before recording.
